# Spread of Virulent Group A *Streptococcus* Type *emm59* from Montana to Wyoming, USA

**DOI:** 10.3201/eid2004.130564

**Published:** 2014-04

**Authors:** Christopher C. Brown, Randall J. Olsen, Nahuel Fittipaldi, Monica L. Morman, Peter L. Fort, Robert Neuwirth, Mohammed Majeed, William B. Woodward, James M. Musser

**Affiliations:** Campbell County Memorial Hospital, Gillette, Wyoming, USA (C.C. Brown, M.L. Morman, P. Fort, R. Neuwirth, M. Majeed, W.B. Woodward);; Houston Methodist Research Institute, Houston, Texas, USA (R.J. Olsen, J.M. Musser);; Houston Methodist Hospital System, Houston (R.J. Olsen, J.M. Musser);; Public Health Ontario, Toronto, Ontario, Canada (N. Fittipaldi);; University of Toronto, Toronto (N. Fittipaldi)

**Keywords:** group A Streptococcus, GAS, bacteria, emm59, streptococci, invasive disease, virulence, genome sequencing, epidemiology, necrotizing fasciitis, Montana, Wyoming, United States

## Abstract

Full-genome sequencing showed that a recently emerged and hypervirulent clone of group A *Streptococcus* type *emm59* active in Canada and parts of the United States has now caused severe invasive infections in rural northeastern Wyoming. Phylogenetic analysis of genome data indicated that the strain was likely introduced from Montana.

Strains of group A *Streptococcus* (GAS) type *emm59* historically have not been commonly associated with invasive infections. However, a striking increase in the frequency and severity of invasive infections caused by type *emm59* strains recently occurred in Canada (*1*).

Four of the authors (M.L.M., P.L.F., R.N., and M.M.) cared for 4 patients whose cases were temporally clustered and who had severe invasive infections caused by GAS in northeastern Wyoming, USA. Of the 4 case-patients, 2 were directly linked. Case-patient 4 contracted invasive infection from a close-contact family member (case-patient 2). Case-patient 3 was a physician who had cared for case-patients 1 and 2. These infections occurred in an area close to where a cluster of 6 invasive *emm59* infections occurred in Montana in 2010 (*2*). This fact led us to determine whether genetically related *emm59* strains were responsible. Full-genome sequencing confirmed this hypothesis (Figure).

**Figure:**
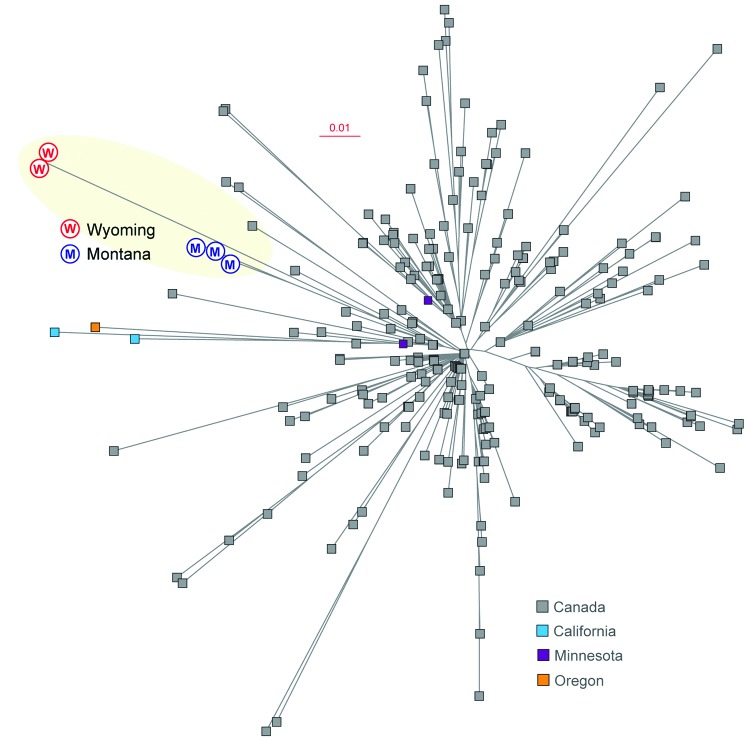
Inferred genetic relationships among group A *Streptococcus* *emm59* strains on the basis of 773 concatenated single nucleotide polymorphisms identified by genome sequencing. Strains from Montana (M) and Wyoming (W), USA, are shown in blue and red, respectively. Strains from Canada, and from California, Minnesota, and Oregon, USA, are shown for reference. Scale bar indicates nucleotide substitutions per site.

## The Cases

### Case-patient 1

A 19-year-old otherwise healthy man had generalized body aches, mild confusion, and cough and was treated symptomatically as an outpatient. The next day, he was found unconscious at his home. In the emergency department, he had hypoxemia, metabolic acidosis, renal failure, leukopenia with 34% bands, and severe unilateral pneumonia. Coagulopathy and sepsis-induced cardiomyopathy with global hypokinesia and reduced ejection fraction developed. He was discharged after a complicated hospital stay. GAS isolated from his throat and blood were discarded but a convalescent-phase serum sample was strongly reactive in a GAS-specific ELISA.

### Case-patient 2

On day 13 after disease onset in case-patient 1, a 78-year-old man with multiple medical problems was hospitalized with cellulitis of the left upper arm. An intravenous line had been inserted into the arm during a cardiac-related hospital admission 4 days earlier. Blood cultures grew GAS. Intravenous vancomycin was administered, and he was discharged and followed up as an outpatient. The following morning, he was found obtunded at home. He had hypoxemia, hypotension, new acute renal failure, and new full-thickness skin necrosis with bullae, mottling, and ecchymoses of the affected arm. He was hospitalized, given broad-spectrum antimicrobial drugs, and provided fluid and pressor support. Imaging studies showed evidence of necrotizing fasciitis. The affected arm was amputated and necrotizing fasciitis was confirmed. He died within 48 hours after hospitalization.

### Case-patient 3

Nineteen days after case-patient 1 was treated and 2 days after case-patient 2 died, generalized chills, profound fatigue, and fever of 102°F developed in a 46-year-old man (physician) who cared for case-patients 1 and 2. Within 24 hours, he had tender cervical lymphadenopathy. At hospitalization, he had normal vital signs tender, left-sided lymphadenopathy, and a leukocyte count of 12,000 cells/μL with 26% bands. He was given broad-spectrum, intravenous, antimicrobial drugs. Imaging studies showed major cervical lymphadenopathy. Blood cultures were negative for bacteria, and a throat culture was not obtained. His recovery was slow and protracted. Convalescent-phase serum analyzed by ELISA was positive for GAS-specific antibodies.

### Case-patient 4

Twenty-five days after the onset of illness in case-patient 1, an inflamed papule developed on the right ring finger of a 46-year-old woman who lived with case-patient 2. The papule showed improvement after treatment with oral antimicrobial drugs. Five days later, malaise, runny nose, anorexia, exertional dyspnea, and pain in the proximal region of the left lower leg developed without associated physical findings. Her symptoms worsened over several days. A 10-cm area of ecchymosis on the thigh and major pain on palpation throughout the leg developed that was out of proportion to visual findings. In the emergency department, she had tachypnea, tachycardia, hypotension, and a normal body temperature. Laboratory studies showed acute renal failure and a leukocyte count of 20,000 cells/μL with neutrophilia but no bands. The patient was transferred to a tertiary care facility because of a presumptive diagnosis of necrotizing fasciitis. She had a complicated hospital course that included amputation of the lower left leg and 2 subsequent surgical extensions. Results of histopathologic analysis of the amputated leg were consistent with those for necrotizing fasciitis. The patient survived.

Strains from case-patients 2 and 4 were sent to Houston Methodist Hospital for genome sequencing to a 65-fold depth of coverage by using a MiSeq Personal Sequencer Instrument (Illumina, San Diego, CA, USA). The 2 *emm59* case strains differed from each by only a 1-nt deletion. Genome data for the 2 sequenced strains have been deposited in the short-read archive of the National Center for Biotechnology Information (Bethesda, MD, USA) under accession nos. SAMN01991041 and SAMN01991042.

Comparison of the genomes of the strains from Wyoming with hundreds of *emm59* genomes (*2**–**4*), determined that the case organisms were most closely related to strains that caused a cluster of 6 invasive infections in rural south central Montana in 2010 (*2*). The *emm59* strains from Wyoming and Montana differed from each another by only 15 single-nucleotide polymorphisms, including 1 in the *covS* gene, which encodes the sensor kinase partner of a key 2-component signal-transduction system (*5*). Structural changes or inactivation of *covR* or *covS* genes can result in up-regulation of up to ≈15% of the GAS transcriptome and increased virulence (*6**–**9*).

Because these cases in Wyoming were temporally and geographically clustered and were caused by closely related genetic variants of the unusually virulent *emm59* clone, an epidemiologic survey was conducted in an effort to identify a reservoir of the case clone. For the first part of the study, 579 cultures were obtained from in-hospital and out-of-hospital contacts, staff, environmental surfaces, and community throat and wound infections. These cultures were obtained from 168 hospital and clinic employees, 343 hospitalized and ambulatory patients and community contacts of the GAS-infected patients, and 68 hospital and community environmental surfaces. These cultures yielded 39 GAS strains, none of which were *emm59*.

The second part of the study included a convenience sample of 17 GAS strains obtained from patients with pharyngitis or skin infections in the geographic area in and around the community where the invasive infections occurred. None of these 17 strains were *emm59*. Many of the *emm* types commonly causing infections in the United States (*10*) (www.cdc.gov/abcs/pathogens/pathogen-links.html) were identified in this sample of 56 GAS strains, including *emm1*, *emm2*, *emm3*, *emm4*, *emm12*, and *emm28*. This result is consistent with the striking lack of *emm59* strains among strains isolated from patients who had pharyngitis during a large survey in Canada (*11*).

## Conclusions

One of the authors (J.M.M.) suggested that the recent and remarkable change in the epidemiologic and virulence behavior of *emm59* GAS strains in Canada and the United States warranted increased attention by public health authorities and infectious diseases practioners (*2*). We document that an unusually virulent *emm59* clone has now emerged to cause severe infections and 1 death in rural northeastern Wyoming. The *emm59* strains have recently emerged as the dominant cause of invasive GAS cases in Minnesota (www.cdc.gov/abcs/reports-findings/survreports/gas12.html), which further illustrates the ability of this clone to successfully spread to other geographic areas and cause abundant infections.

One limitation of our study is that we had isolates available from only 2 of the 4 case-patients. However, extensive review of hospital records identified only 1 case of GAS necrotizing fasciitis in the past 5 years, which underscores the otherwise rarity of these episodes in northeastern Wyoming. Thus, we believe we can reasonably hypothesize that all 4 case-patients were infected with clonally related *emm59* GAS strains.

Given the speed and modest cost of full-genome sequencing and its role in human health, we remain interested in studying the spread of this clone. Persons responding to clinical situations that might warrant strain genome sequencing should contact the corresponding author.
